# Translational-circular scanning for magneto-acoustic tomography with current injection

**DOI:** 10.1186/s12938-016-0125-x

**Published:** 2016-01-27

**Authors:** Shigang Wang, Ren Ma, Shunqi Zhang, Tao Yin, Zhipeng Liu

**Affiliations:** Institute of Biomedical Engineering, Chinese Academy of Medical Sciences & Peking Union Medical College, Tianjin, 300192 China; College of Radiology, Taishan Medical University, Taian, 271016 China

**Keywords:** Magneto-acoustic tomography with current injection, Translational-circular scanning, Acoustic-source imaging

## Abstract

**Background:**

Magneto-acoustic tomography with current injection involves using electrical impedance imaging technology. To explore the potential applications in imaging biological tissue and enhance image quality, a new scan mode for the transducer is proposed that is based on translational and circular scanning to record acoustic signals from sources.

**Methods:**

An imaging algorithm to analyze these signals is developed in respect to this alternative scanning scheme. Numerical simulations and physical experiments were conducted to evaluate the effectiveness of this scheme. An experiment using a graphite sheet as a tissue-mimicking phantom medium was conducted to verify simulation results. A pulsed voltage signal was applied across the sample, and acoustic signals were recorded as the transducer performed stepped translational or circular scans. The imaging algorithm was used to obtain an acoustic-source image based on the signals.

**Results:**

In simulations, the acoustic-source image is correlated with the conductivity at the sample boundaries of the sample, but image results change depending on distance and angular aspect of the transducer. In general, as angle and distance decreases, the image quality improves. Moreover, experimental data confirmed the correlation.

**Conclusion:**

The acoustic-source images resulting from the alternative scanning mode has yielded the outline of a phantom medium. This scan mode enables improvements to be made in the sensitivity of the detecting unit and a change to a transducer array that would improve the efficiency and accuracy of acoustic-source images.

## Background

Medical imaging technology provides structural and pathological information on human tissue and plays an important role in clinical diagnostics. X-ray tomography and Computed tomography (CT) use the absorption coefficient of X-rays in human tissue and organs, however, X-rays can cause damage to the body [1]. Magnetic resonance imaging analyses magnetic resonance signals from human tissue [2]; however, magnetic resonance imaging equipment is relatively expensive. Ultrasound imaging exploits differences in acoustic impedance of tissue, although the contrast is not good for soft tissue. These imaging technologies are better for imaging structures and helping in disease diagnosis. However, they cannot distinguish functional changes in biological tissue. Electrical impedances of tissue and organs vary in diseased conditions [3]. Therefore, imaging approaches to obtain the electrical properties of biological tissue could be helpful in early diagnosis, and, for this reason, have attracted considerable interest in recent years.

Among these electrical impedance imaging techniques, electrical impedance tomography (EIT) [4–7] uses surface electrodes to pass a current and measure the resultant surface voltage for reconstructing images. This technique enables real-time imaging and is low-cost and safe; however, the passed current cannot flow deep into tissue because of electrical shielding. Moreover, limited surface measurements lead to the ill-posed inverse problem and low spatial resolution. To mitigate shielding, magnetic induction tomography [8, 9] has been developed and involves using an excitation magnetic field to induce current in the tissue that generates a secondary magnetic field measured by external sensing coils. An image of conductive media can be constructed from the induced secondary magnetic field. Whereas magnetic induction tomography overcomes the shortcomings of EIT derived from shielding, this secondary magnetic field is invariably too weak to be separated from the excitation magnetic field, and hence limits the spatial resolution of the constructed image. To obtain high spatial resolution, magnetic resonance electrical impedance tomography [10, 11] is a technique whereby current is passed through a pair of electrodes, and a magnetic resonance scanner measures the magnetic flux density in the intervening tissue. Compared with EIT, magnetic resonance electrical impedance tomography improves the spatial resolution of the constructed image but requires a relatively expensive magnetic resonance system. Moreover, a large electric current must be applied to obtain signals of an acceptable signal-to-noise ratio (SNR).

Imaging modality coupling multi-physical field is a new functional imaging method for tissue, and introduces an alternative way to image electrical impedance. Combining the technologies of EIT and USI, magneto-acoustic tomography (MAT) [12, 13] uses differences in conductivity of biological tissue as the imaging target. Conductive tissue includes the structural boundaries of organs and tissue having variation in conductivity arising from functional changes. In the excitation mode, MAT separates into two classes: MAT with magnetic induction (MAT-MI) and MAT with current injection (MAT-CI). The former was recently proposed by He Bin’s group to image the distribution of conductivity with high spatial resolution [14, 15]. In brief, the sample is placed in static and time-varying magnetic fields. The time-varying magnetic field induces an eddy current in the sample that consequently emits ultrasonic waves as a result of the Lorentz force. These waves are detected using transducers located around the sample. With these signals, an image of conductivity distribution can be constructed using appropriate algorithms [16–20].

A few groups are working on MAT-CI [21, 22]. Indeed, its method is similar to MAT proposed earlier and studied by Towe [12]. MAT-CI requires a small and safe current passing through the sample. In contrast, MAT-MI requires voltages of a few kV to generate a pulsed magnetic field that induces the eddy currents in the biological tissue [23]. Indeed, MAT-CI requires no pulse-field generator, and hence is easily produced and cheap to implement. Nevertheless, research on MAT-CI is still in its preliminary stage.

The image quality of MAT-CI depends on the detected ultrasound signals, which are related to the gain map distribution of the transducer and scanning scheme. In the traditional circular scanning scheme, the transducer moves around the sample which is placed in a region relatively far from the transducer. In this way, a non-focused transducer can be used but the sensitivity of the MAT-CI system is low. Moreover, the step angle in the traditional circular scanning mode should be small. If the angular increment is large, the constructed image will be distorted because the acoustic signals will be poor [18]. To improve the sensitivity of the detected signals and to understand the processes involving the transducer array in MAT-CI, we propose an alternative scanning scheme called the translational-circular scan (TC-scan) mode and performed simulations and experiments on a graphite model.

## Methods

### MAT-CI principle

**Fig. 1:**
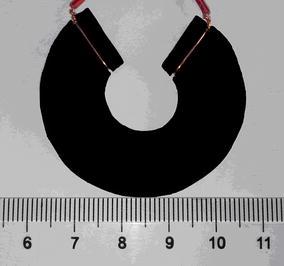
Photo of the graphite slice model

In MAT-CI, the sample is placed in a static magnetic field and a pulse current is passed through it. Because of the static magnetic field, the current is subject to a Lorentz force, and the particles within the sample begin to vibrate emitting acoustic waves. Moreover, the characteristics of the acoustic signal are determined by the applied voltage and the conductivity of the sample. After analyzing the acoustic signals, we can obtain the variation of conductivity within the sample and construct an image. In these experiments, the width of the pulse current was an order of 10^−6^ s, and the wavelength of the electromagnetic wave was an order of 10^2^ m. We fabricated a sheet of graphite (Fig. 1) of a certain thickness, and a size of roughly 10^−2^ m, much smaller than the wavelength of the electromagnetic wave. Hence, the conditions of an electro-quasistatic field hold within the sample [24]. Moreover, the frequency of the current, being an order of 1 KHz [25], can be ignored in this study because it is much smaller than the input current.

If a phantom medium has isotropic conductivity and a regular shape such as a disk, the basic equations governing the electro-quasistatic field are:

1 $$ \nabla \cdot \varvec{J} = 0, $$

2 $$ \nabla \times \varvec{E} = 0. $$

Here bold variables refer to vectors. However, if the shape of the phantom medium or the boundary of conductivity is irregular, the divergence of ***J*** is nonzero.

From Eq. (2), electric field ***E*** can be represented by an electric scalar potential *φ*,

3 $$ \varvec{E} = - \nabla \phi . $$

In an isotropic and homogeneous medium, Ohm’s law is

4 $$ \varvec{J} = \sigma \varvec{E} . $$

With Eqs. (3) and (4) substituted into Eq. (1), we obtain the Laplace equation:

5 $$ \nabla^{2} \phi = 0 . $$

The conditions at the boundary of the medium are [24]

6 $$ \phi_{1} = \phi_{2} , $$

7 $$ \sigma_{1} \frac{{\partial \phi_{1} }}{{\partial \varvec{n}}} = \sigma_{2} \frac{{\partial \phi_{2} }}{{\partial \varvec{n}}} , $$

where ***n*** is the unit normal vector at the boundary. Combining Eqs. (5)–(7) and the field boundary conditions, the electric potential can be solved. The current density in the sample can then be computed using Eqs. (3) and (4).

The acoustic wave equation for MAT-CI can be written [26] as

8 $$ \nabla^{2} p(\varvec{r},t) - \frac{1}{{c^{2} }}\frac{{\partial^{2} }}{{\partial t^{2} }}p(\varvec{r},t) = \nabla \cdot [\varvec{J}(\varvec{r},t) \times \varvec{B}] , $$

where ***r*** is the location of the acoustic source, *p* acoustic pressure, *c* acoustic velocity in tissue, ***J*** injected current density, ***B*** static magnetic field flux density, and ∇^2^ and ∇ are the Laplacian operator and divergence operator, respectively. The term on the right-hand side of Eq. (8) represents the vibration source, which Zhang et al. derived in detail [26]. After introducing Green’s function, we obtain an analytical solution of Eq. (8)

9 $$ p(\varvec{r} ',t) = - \frac{1}{4\pi }\iiint\limits_{\varSigma } {d\varvec{r}\nabla \cdot [\varvec{J}(\varvec{r},t) \times \varvec{B}]\frac{{\delta (t - |\varvec{r} '- \varvec{r}|/c)}}{{|\varvec{r} '- \varvec{r}|}}} . $$

However, every transducer has its own gain map distribution which is determined by its structure. Assume *w*(***r***, ***r′***) is the weight factor for the transducer receiving signals; that is, *w*(***r***, ***r*′**) is the acoustic gain map with ***r*** denoting the position of the source of the acoustic signal, and ***r*′** the position of the acoustic transducer. Depending on the transducer’s characteristics, the signal of the acoustic vibration emitted from ***r*** and received by the transducer at ***r*′** can be written

10 $$ p_{1} (\varvec{r},\varvec{r}') = \nabla \cdot [\varvec{J}(\varvec{r},t) \times \varvec{B}] \cdot w(\varvec{r},\varvec{r}') . $$

Considering the focus region Σ of the focused transducer and time delay of the acoustic propagation, signals received by the transducer can be written as

11 $$ p_{2} (\varvec{r}',t) = \iiint\limits_{\varSigma } {dr\nabla \cdot [\varvec{J}(\varvec{r},t) \times \varvec{B}] \cdot w(\varvec{r},\varvec{r}') \otimes \delta (t - |\varvec{r}' - \varvec{r}|/c)} , $$

where ⊗ is the convolution operator. In Eq. (11), the convolution function is an impulse located at *r*. The acoustic pressure of an acoustic source point is *p*_1_, so all of the sound source point is *p*_2_, that is, the integral of *p*_1_ is *p*_2_. Taking into account the impulse response *h*_1_(*t*) of the transducer, we obtain

12 $$ p(\varvec{r}',t) = \iiint\limits_{\varSigma } {dr\nabla \cdot [\varvec{J}(\varvec{r},t) \times \varvec{B}] \cdot w(\varvec{r},\varvec{r}') \otimes h_{1} (t - |\varvec{r}' - \varvec{r}|/c)} . $$

### Translational-circular scan mode

In the TC-scan mode, the transducer is placed at a location close to the sample. With the sample located at the focal region of the transducer, better characteristics are obtained there than in the non-focused region. The TC-scan mode yields good sensitivity.

**Fig. 2:**
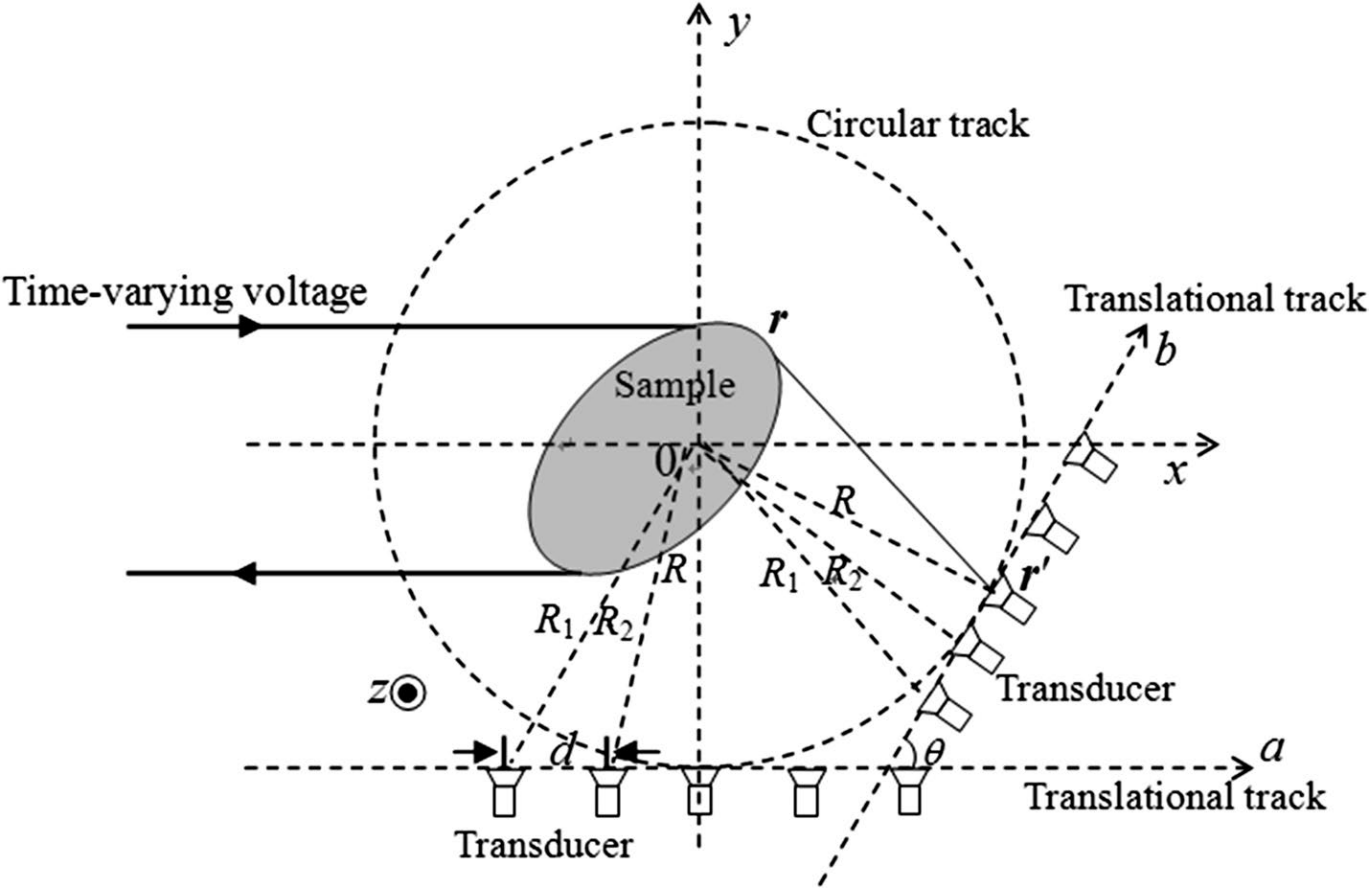
Schematic diagram of translational-circular scan mode for magnetoacoustic tomography with current injection. The dot represents that the direction of the static magnetic field is the positive direction of the z axis

The procedures associated with the TC-scan mode are described (Fig. 2). Initially, the transducer is located at one end of straight line *a.* After receiving an acoustic signal, the transducer moves a step distance *d* along *a*, and receives another signal. The transducer is incrementally moved along *a* in steps of *d*, registering acoustic signals until it reaches the other end of *a*. The length of line *a* should be equal to or longer than the maximum diameter of the sample. Next, the line *a* is rotated with angular increment *θ* about an axis centered within the sample at radius *R*. The transducer repeats the procedure as before. In this way, *a* rotates and the transducer receives signals until the line *a* returns to its initial position. At this point, signal acquisition is complete.

Acoustic-source imaging is the inverse problem to the acoustic field signal. To construct an accurate acoustic-source image, the acoustic signal *p*(***r***′, *t*) is deconvoluted to obtain the actual acoustic signal *p*_2_(***r***′, *t*), which, fortunately, can be achieved using a Wiener filter [22]. The scanning procedure (Fig. 2,) can be separated into translational and circular processes. Indeed, at every rotational angle, every position of the transducer has a distinct radius (Fig. 2). For example, both the radii of the first transducer at line *a* and *b* are *R*_1_, and both the radii of the second transducer at line *a* and *b* are *R*_2_. The TC-scanning procedure is the combination of several traditional circular scanning of different radii.

In acoustic-source imaging, the sub-image at each rotational angle *k*_1_*θ* (*k*_1_ refers to the number of rotational increments of line *a*) is established first. The spatial characteristic of the acoustic transmission were discussed in regard to the previous problem. For any rotational angle *k*_1_*θ*, there are the associated translational increments of the transducer in the new scanning scheme. Taking into account the properties of the focused transducer, the detected acoustic signals mainly come from acoustic sources in the focus region. To construct the sub-image, we estimate the acoustic source value in the region, where the middle line is set as the beam axis of the transducer, and the width of the region is *d*. Assuming cylindrical coordinates because of the scan configuration [27], the algorithm is applied to the focused region and the acoustic intensity *I*_*k2*_(***r***) can be obtained as follows:

13 $$ {\text{I}}_{K2} (r )\approx \frac{ - 1}{{2\pi {\text{c}}^{3} }}\iint\limits_{\varSigma } {{\text{ds}}\sqrt {1 - \frac{{ ( {\text{z}}_{ 0} -{\text{  z)}}^{2} }}{{\left| {r - r^{\prime}} \right|^{2} }}} \times{ {\text{p}}_{K2}}^{\prime \prime } \left( {r^{\prime},\frac{{|r^{\prime} - r|}}{\text{c}}} \right)} , $$

where *p*_*k*2_″(***r***′, *t*) is the second-order derivative of the *k*_2_th transducer pressure, and *I*_*k2*_(***r***) is obtained from the *k*_2_th transducer and provides part of the information of the sub-image. In this way, we obtain the acoustic-source sub-image *I*_*k*1*θ*_(***r***) for rotational angle *k*_1_*θ*,

14 $$ I_{k1\theta } (r) = \sum {I_{k2} (\varvec{r})} . $$

Eq. (14) represents the acoustic-source sub-image of the *k*-incremented angle, and provides only part of the acoustic-source information. To obtain the complete image, calculations from all sub-images *I*_*k*1*θ*_(***r***) must be combined. This requires a summation of all sub-images from the different rotational angles *k*_1_*θ* and averaging,

15 $$ I(r) = \frac{{\sum {I_{k1\theta } (r)} }}{n(r)} , $$

where *n*(***r***) is the number of iterations at ***r***. After the acoustic-source information is derived, the distribution in conductivity can be constructed using electromagnetic theory.

### Simulation method

To study the TC-scan scheme of MAT-CI, simulations and experiments were performed using a sheet of graphite of a certain thickness. Numerical solutions of the graphite model were obtained using COMSOL Multiphysics modeling software (Comsol, Stockholm, Sweden); simulations of the acoustic pressures and acoustic-source distribution were performed using MATLAB. In addition, we applied an experimental MAT-CI system to measure the acoustic signals.

**Fig. 3:**
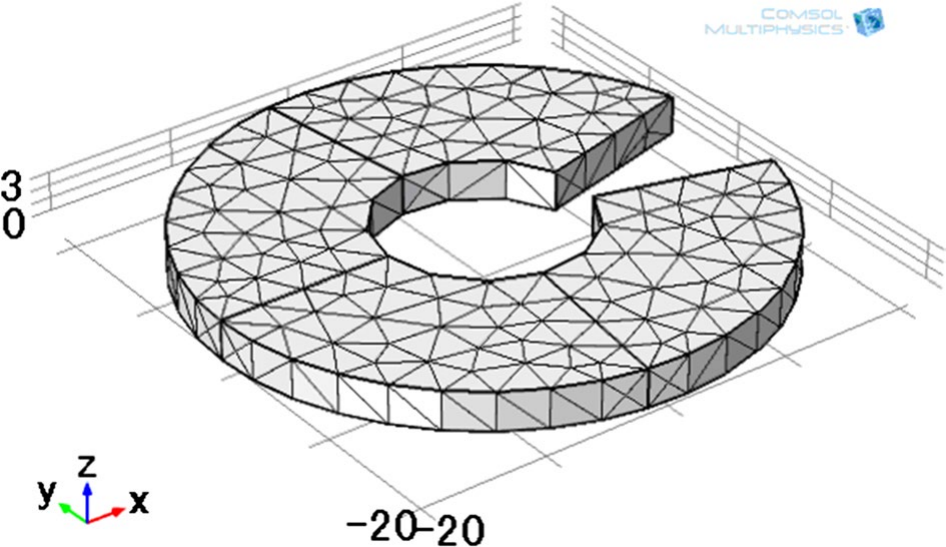
Model and mesh of the graphite slice (outer diameter 43 mm; inner diameter16 mm)

**Fig. 4:**
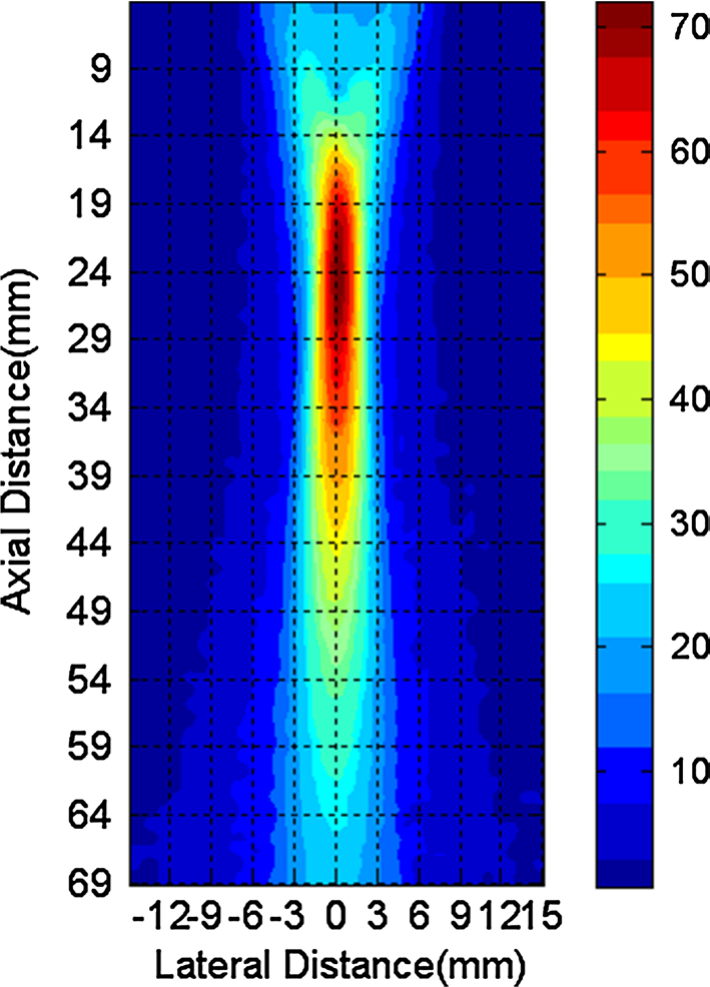
Gain map of the customized transducer. The focused region dimension of the transducer is about 44 mm long (13–59 mm) and about 6 mm wide (−3–3 mm)

Computer simulations using COMSOL Multiphysics were performed in the frequency domain, and the parameters were set in accordance with the experimental setup. To aid the numerical calculation of the conductivity model, the excitation signal was set to 5 V. The graphite model was meshed with free split tetrahedral shapes (Fig. 3), the maximum and minimum element sizes being 0.4 mm and 0.004 mm, respectively. The graphite model was 3-mm thick; its outer and inner diameters were 43 mm and 16 mm, respectively; the conductivity for the graphite sheet was set at 10^5^ S/m. Because the propagation speed of the acoustic waves in the tissue is similar to that in water, i.e., about 1500 m/s, the velocity in the simulation was set to 1500 m/s. The flux density of the static magnetic field was 0.4 T. Figure 4 shows the gain map distribution of the transducer (center frequency 1.06 MHz and diameter 20 mm) used in simulations. The transducer is focused, and the focused region was about 44 mm (13–59 mm) long and about 6 mm (−3–3 mm) wide (Fig. 4).

In scanning, line *a* was 45 mm long with a rotational radius of 33 mm. By maintaining step distance *d* constant, the variation of imaging results with angular increments was analyzed. Similarly, by keeping step angle *θ* constant, the variation of the imaging results with radial increments was analyzed. The mean squared error (MSE) and correlation coefficient (CC) were used to evaluate the imaging quality,

16 $$ MSE = \frac{{\mathbf{1}}}{MN}\sum\limits_{i = 1}^{M} {\sum\limits_{j = 1}^{N} {(X(i,j) - Y(i,j))^{2} } } , $$

17 $$ CC = \frac{{\sum\nolimits_{i = 1}^{M} {\sum\nolimits_{j = 1}^{N} {(X(i,j)} } - \overline{{\mathbf{x}}} ) \cdot (Y(i,j) - \overline{{\mathbf{y}}} )}}{{\sqrt {\sum\nolimits_{i = 1}^{M} {\sum\nolimits_{j = 1}^{N} {(X(i,j)} } - \overline{{\mathbf{x}}} )^{2} \cdot \sum\nolimits_{i = 1}^{M} {\sum\nolimits_{j = 1}^{N} {(Y(i,j)} } - \overline{{\mathbf{y}}} )^{2} } }} , $$

where $\overline{{\mathbf{x}}} = \frac{1}{MN}\sum\nolimits_{i = 1}^{M} {\sum\nolimits_{j = 1}^{N} {X(i,j)} }$, $\overline{{\mathbf{y}}} = \frac{1}{MN}\sum\nolimits_{i = 1}^{M} {\sum\nolimits_{j = 1}^{N} {Y(i,j)} }$. *X*(*i*, *j*) and *Y*(*i*, *j*) are the original image data and constructed image data, respectively. MSE and CC assessed the similarity in spatial distribution between the constructed and original images. Image reconstruction is more accurate when the value of MSE is small and the value of CC is large.

### Experimental method

Following the principles of MAT-CI, an experimental setup was developed. Because the acoustic velocity in biological tissue is similar to that in water, water was used as the acoustic coupling agent to reduce scattering and reflection of acoustic waves. The sample and transducer were both immersed in water. The excitation signal was generated using a signal generator (AFG3252, America Tektronix Inc., Beaverton, OR, USA). After amplifying the pulsed sine signal using a power amplifier (HSA4101, Japan NF Inc., Yokohama, Japan), it was applied across the sample. A stepper motor (Customized from the Institute of Electrical Engineering, Chinese Academy of Sciences; center frequency 1.06 MHz and diameter 20 mm, the focused region was about 44 mm (13–59 mm) long and about 6 mm (−3–3 mm) wide) performed the translational and rotational increments of the stage holding the transducer in scanning the sample; the transducer registered the acoustic signals and converted them into electrical signals. The electrical signal was magnified by an amplifier (NF5307; Japan NF Inc.) and displayed on an oscilloscope (MS04104; America Tektronix Inc.). The amplified signal was also sampled by an acquisition card and digitally stored on a computer. To keep the excitation and received signals synchronized, the computer recorded the excitation signal from the signal generator at the same time.

The parameters for the graphite model were the same in the experiments as in the simulations. Using the above experimental devices, the experiments using a graphite sheet as a tissue-mimicking phantom were implemented. The strength of the static magnetic field was 0.4 T; the length and rotational radius of the transducer’s translational line were 45 mm and 33 mm, respectively. The signal generator output was a sinusoidal pulse 1 µs-wide with a peak–peak value of 5 V. The signal was then amplified by 100 and loaded on the sample. The acoustic signals received by the transducer were magnified 100 times by the preamplifier and averaged 1024 times to reduce noise and digitally stored. An averaging filter of 1024 sinusoidal pulses, each with a repeating frequency of about 1000 Hz to remove the random noise for every averaged sample, was used. The sampling frequency of the acquisition card was 15 MHz. The sample size was the same as the simulation parameters (Fig. 1).

## Results and discussion

### Simulation results

**Fig. 5:**
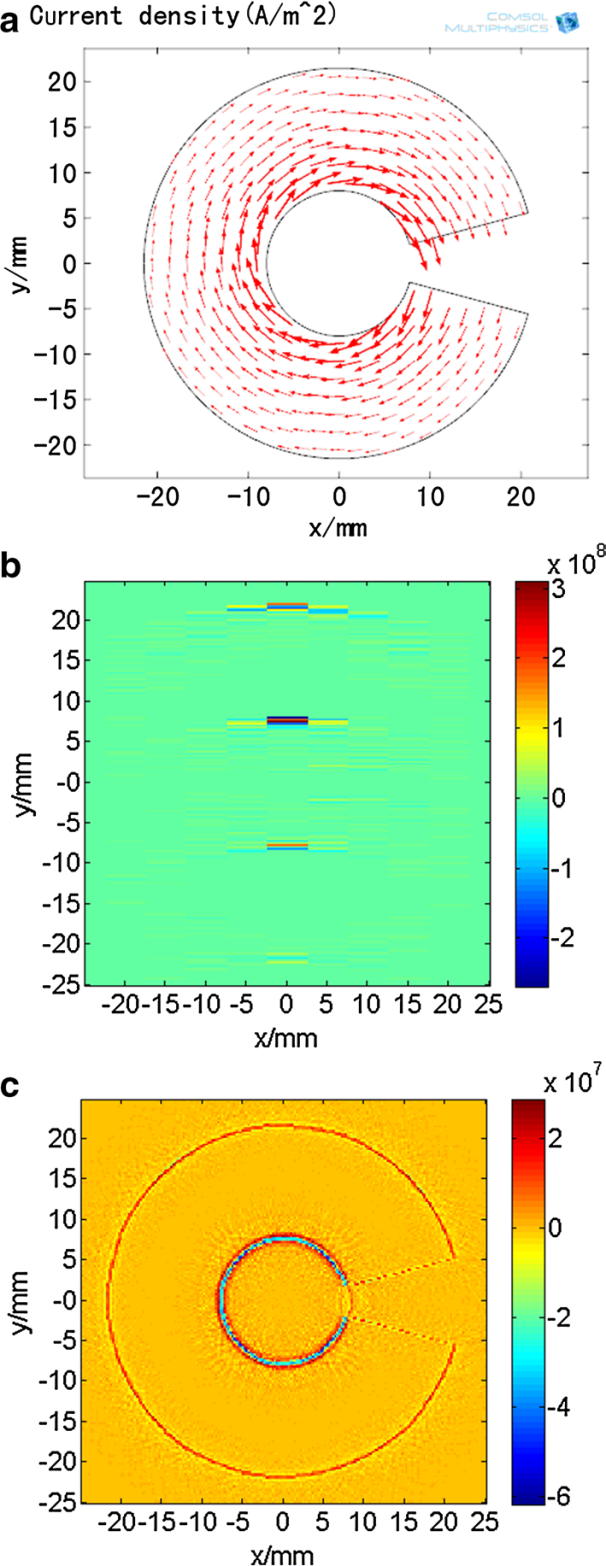
Simulation results in the Z = 1.5-mm plane. **a** Current density distribution. The vector arrows represent the XY components of the current density field. **b** Reconstructed sub-image at rotational angle *k*_1_ *θ* = 0°. **c** Reconstructed acoustic source image at step angle *θ* = 3.6°and step distance *d* = 3 mm

The data for conductivity and current density in the Z = 1.5 mm plane was derived using COMSOL Multiphysics, and simulations were performed with the above-stated conditions. The results of the current density distribution (Fig. 5a) show that the inner current was greater than the outer current. With step angle set at *θ* = 3.6° and the step distance at *d* = 3 mm, the acoustic-source sub-image for rotational angle *k*_1_*θ* = 0° (Fig. 5b) provides a part of the information about acoustic sources; the acoustic-source image (Fig. 5c) shows a similar outline and size to the optical image of sample. This implies that the acoustic-source image from the conductivity of the sample correlates strongly with its boundaries

**Fig. 6:**
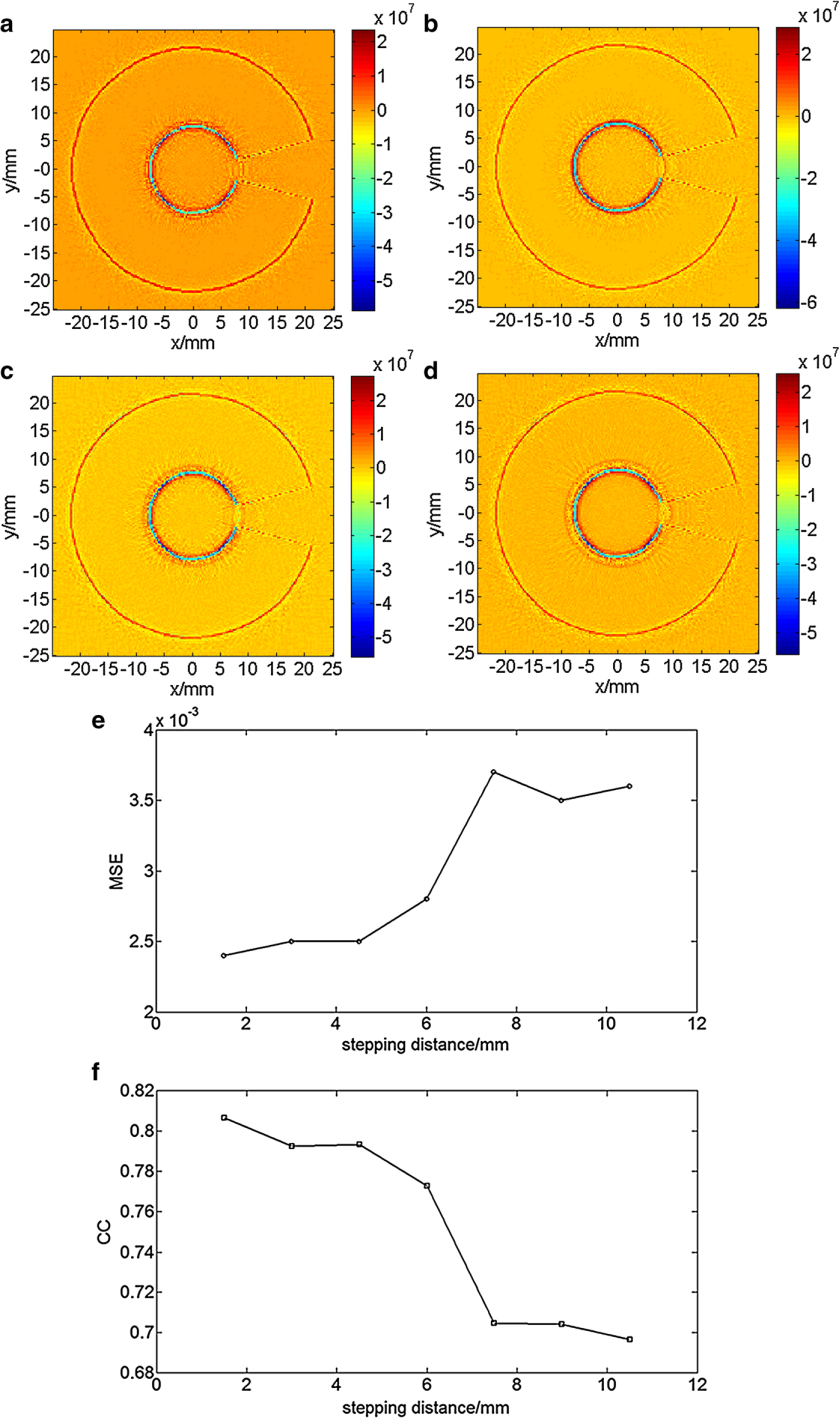
Reconstructed results at step angle *θ* = 3.6° in Z = 1.5-mm plane. **a** Reconstructed acoustic distribution at step distance *d* = 1.5 mm. **b** Reconstructed acoustic distribution at step distance *d* = 3 mm. **c** Reconstructed acoustic distribution at step distance *d* = 4.5 mm. **d** Reconstructed acoustic distribution at step distance *d* = 6 mm. **e** Mean Squared Error distribution with a variety of step distances. **f** Correlation Coefficient distribution with a variety of step distances

The imaging results changed with different values for the step distance *d* and step angle *θ*. Thus, the relationship between the image quality and different distances and angles needs to be analyzed. First, with fixed step angle *θ* = 3.6°, images (Fig. 6a–d) were generated for incremental step distances *d* of 1.5, 3, 4.5, and 6 mm, respectively. From the four images, the boundaries become blurred and the outline fades with increasing step distance. With the same fixed step angle *θ* = 3.6°, the variation of MSE and CC with step distance (Fig. 6e, f, respectively) show as expected that MSE increases and CC decreases with increasing distance. However, at distances greater than 7.5 mm, both MSE and CC remain level.

**Fig. 7:**
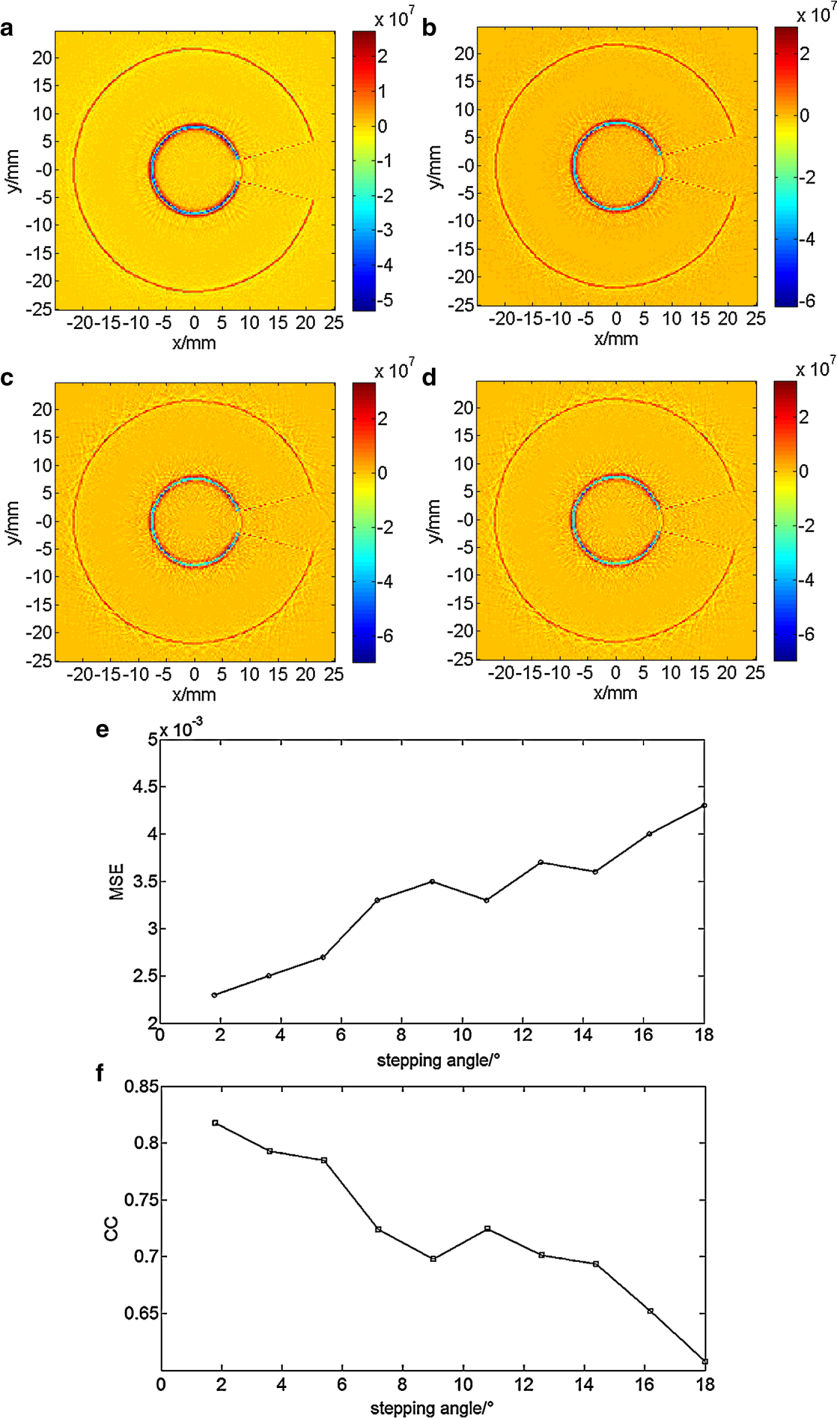
Reconstructed results at step distance *d* = 3 mm in Z = 1.5-mm plane. **a** Reconstructed acoustic distribution at step angle *θ* = 1.8°. **b** Reconstructed acoustic distribution at step angle *θ* = 3.6°. **c** Reconstructed acoustic distribution at step angle *θ* = 7.2°. **d** Reconstructed acoustic distribution at step angle *θ* = 14.4°. **e** Mean Squared Error distribution with a variety of step angles. **f** Correlation Coefficient distribution with a variety of step angles

We performed a similar procedure with fixed step distance of *d* = 3 mm. The various images from the acoustic sources with incremental angular steps of 1.8, 3.6, 7.2 and 14.4° (Fig. 7a–d) show that there are no significant differences for angles less than 7.2°. The outline of the acoustic source is distinguished up to angles of 14.4°. For the same fixed step distance of *d* = 3 mm, the variation of both MSE and CC with increasing angle (Fig. 7e, f) show similarly that MSE increases and CC decreases. This indicates that the image quality depends on step angle.

### Experimental results

**Fig. 8:**
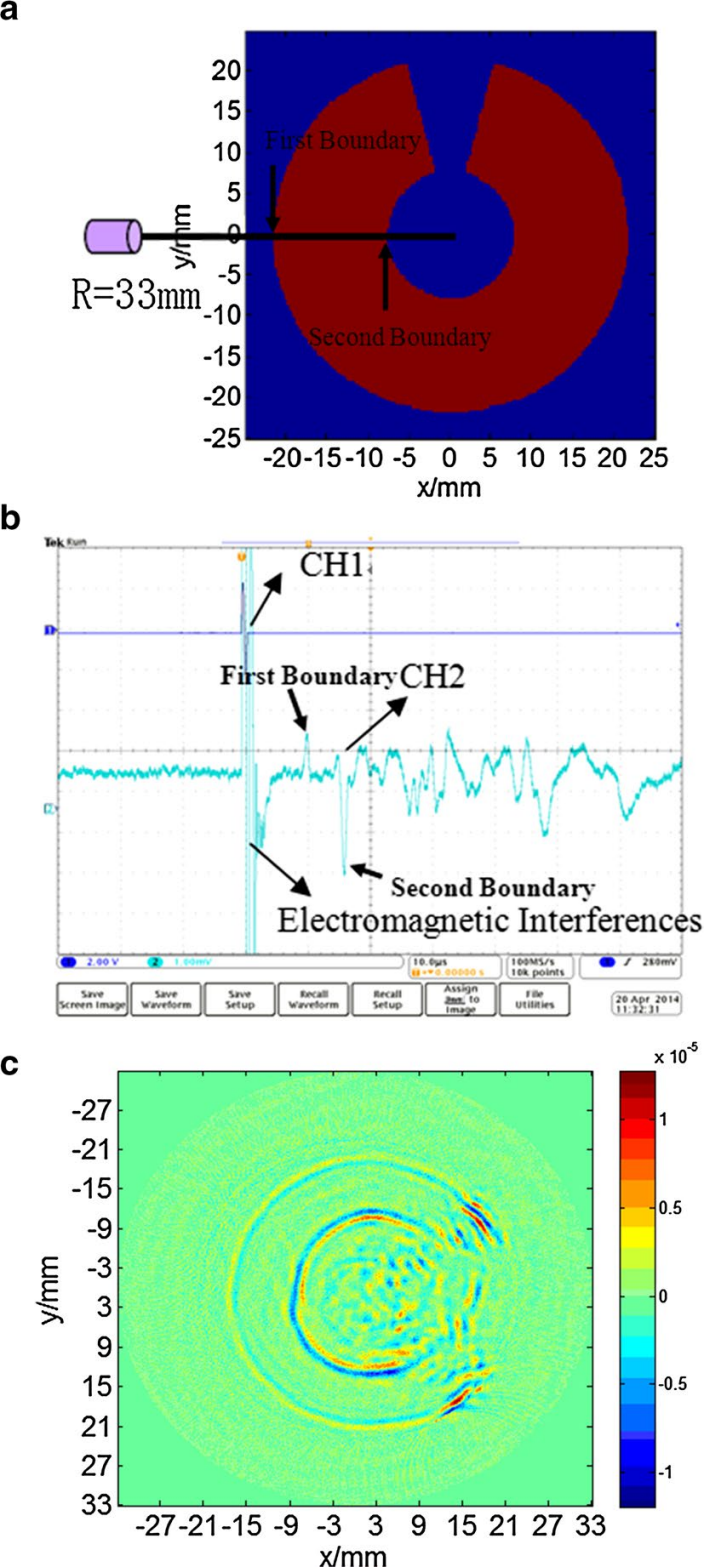
Experimental results. **a** Transducer’s position relative to the graphite slice. **b** Collected acoustic signal from the transducer. **c** Reconstructed image with step distance *d* = 3 mm and step angle *θ* = 3.6°

Using the setup for the MAT-CI system, experiments were performed with the new scanning mode. In the experiments, the phantom medium was a sheet of graphite with outer and inner diameters of 43 and 16 mm, respectively (Fig. 8a). Acoustic wave signals were recorded at step distance *d* = 3 mm and step angle *θ* = 3.6°. The excitation pulse was a sine pulse signal of width 1 μs and peak–peak voltage of 5 V. The sine pulse signal was amplified 100 times using a power amplifier and supplied to the sample, which was in series with a 51 Ω resistor. The acoustic signal detected by the transducer was magnified 100 times by the amplifier and averaged 1024 times. From the position of the transducer (Fig. 8a, b) shows the waveforms of the collected acoustic signal obtained (channel CH2 of the oscilloscope with settings of 2 mV/div, 10 μs/div) and of the excitation voltage (CH1 with settings of 1 V/div, and 10 μs/div). There is obvious noise caused by electromagnetic interference (EMI) in addition to spurious noise in the signals received. The image was constructed (Fig. 8c) using Eqs. (13)–(15); however, because the acoustic wave signal contained considerable noise, visible distortions can be seen. Nevertheless, the resultant image outlines the shape of the phantom medium and is consistent with its optical image (Fig. 1).

### Discussion

Comparing the TC-scan mode with the traditional circular scanning mode, the former involves using the focused region of the transducer where the sensitivity is significantly greater. The intensity in the focused region (Fig. 4) is 70 times greater than outside the focused region. Compared with the B-Scan mode [28], both scanning modes used the focused region of the transducer; however, their scanning processes are different. In the B-Scan mode, the transducer carries out sector scanning at different locations around the object. In the TC-scan mode, the transducer performs translational scans for a preselected set of angles. Recall that TC-scanning was introduced to investigate the response of a transducer array in MAT-CI.

We also performed simulations to compare the performance of TC-scan mode with that of conventional circular-scanning mode. From our imaging results of the acoustic sources, there is significant projection noise in the circular-scan mode, especially with wide step angles. There is some peripheral noise resulting from translating the acoustic transducer in the TC-scan mode. Given the same step angle, the TC-scan mode is superior to the circular-scan mode. Moreover, if the imaged phantom medium has special shapes, for example rectangular, and even if the step angle is small, the image quality is relatively poor in the conventional circular-scan mode; whereas it is relatively good in the TC-scan mode. Naturally, the number of acoustic signals sampling is significantly more in the TC-scan mode than in the conventional circular-scan mode.

Because the methods employed in MAT-CI and MAT are similar, we focused on evaluating the alternative TC-scan mode. This is different to that for MAT-MI, which uses induction, hence the coining of our method as MAT-CI.

In addition, as the magneto-acoustic signal includes considerable noise from electronics and EMI, reducing the noise in the detecting unit of the experimental system is required. Indeed, with large mechanical heterogeneity at the graphite–water interface, many reflections occur, creating differences in acoustic impedance. These acoustic reflections were neglected in the signal processing. At the same time, the electric wires also produced acoustic signals, which were also neglected. Together with denoising algorithms, hardware must be developed to improve the SNR. The peak signals at the first and second boundaries of the graphite model (Fig. 8b) are clearly visible in acoustic signal acquisition experiments (Cf., first and second boundaries of the phantom medium in Fig. 8a).

The peak signals from the third and fourth boundaries were submerged in noise because of reflecting oscillations of the third and fourth boundary signals in the ring. Even when the transducer’s far-field region was used, the TC-scan mode was able to image the phantom medium; this is an additional advantage, and may reduce the sensitivity of the system.

The signal intensity associated with the second conductivity boundary in magneto-acoustic signal was significantly greater than that of the first conductivity boundary (Fig. 8b). This suggests that the current at the second boundary and the vibration intensity are initially greater. The distribution of the current density (Fig. 5a) also supports this conclusion and validates the theory.

Moreover, given the acoustic source ∇∙[***J***(***r***,*t*) × **B**] = [∇ × ***J***(***r***,*t*)]∙**B** + [∇ × **B**]∙***J***(***r***,*t*) = [∇ × ***J***(***r***,*t*)]∙**B** and ***J***(***r***,*t*) **=** ***σE***(***r***,*t*), we have ∇∙[***J***(***r***,*t*) × **B**] = ∇×[***σE***(***r***,*t*)]∙**B** = [***σ***∇ × ***E*** (***r***,*t*) + (∇***σ*)** × ***E***]∙**B**. Hence in the inner conductivity region, we have ∇∙[***J***(***r***,*t*) × **B**] = [***σ***∇ × ***E***(***r***,*t*)]∙**B**. If conductivity is not uniform, ***σ***∇ × ***E***(***r***,*t*) ≠ 0; then there is an acoustic source and the internal conductivity distribution can be imaged. However, if conductivity is uniform, ***σ***∇ × ***E***(***r***,*t*) = 0; then, there is no acoustic source and therefore the internal conductivity distribution cannot be imaged. Because the conductivity distribution of the graphite model was uniform, MAT-CI can only image the conductivity boundary of the graphite model, but not the internal conductivity distribution (see Fig. 8c). Hence, the acoustic sources reside only at the conductivity boundaries, which are precisely what are imaged.

With only images of the acoustic sources at the boundaries, the conductivity distribution image cannot be constructed. This constitutes the inverse problem of the electromagnetic field, and on this issue further research is needed.

Although each acoustic-source image correlates with the outline of graphitic phantom medium (Fig. 8a, b), the size of these images change because of errors caused by velocity inhomogeneity, i.e., the radius of the inner ring had increased. Because the acoustic speed in water (1.5 mm/*μ*s) is different from that in graphite (2 mm/*μ*s), the inner radius observed in the image increases. If the velocity was uniform, this circumstance would not appear. This behavior was also confirmed in an experiment using a copper ring [26].

The acoustic signal depends linearly on the conductivity of the phantom medium, that being a graphite sheet in the experiment. The conductivity of graphite is 100,000 times that of tissue. When using biological tissue as phantom medium, an acoustic signal with an acceptable SNR cannot be obtained. Clearly, the system needs optimizing, for example by increasing surface currents and the intensity of the static magnetic field, or using a preamplifier with a relatively high common mode rejection ratio. This will remain a focus of future work.

Both simulations and experiments were performed in imaging of acoustic sources. However, limited by the finite bandwidth of the transducer, information during the detection of the acoustic wave signal is inevitably lost. Further study on conductivity imaging is required particularly with respect to incomplete signal data. Progress in this field has been reported by MAT-MI researchers [29, 30], but remains to be resolved for MAT-CI.

Although MAT-CI research is still in its early stage, the potential clinical application areas of this method are not limited to the early diagnoses of tumors such as mammary cancer. Benefits might accrue with its application in evaluating or monitoring treatment outcomes of tumors.

## Conclusions

Simulations and experiments using the TC-scan mode of MAT were performed. The simulation results obtained for acoustic-source images from conductivity distributions of a phantom medium reproduced well its boundaries. The experimental results also show good agreement with optical profiles of the phantom medium. The study showed that both the step distance and step angle affect image quality. In general, when the step angle and step distance decreased, the image quality improved.

The particular scanning used in the TC-scan mode has several merits. First, with the phantom medium at the focus region of the transducer, the sensitivity of the detecting unit improves. Second, for future applications, a transducer array could be used to improve efficiency and accuracy. The new scan scheme would only need rotations of the transducer array. This proposed mode is a promising method to advance clinical applications of MAT-CI.
